# La tuberculose sternale: à propos de 2 cas

**Published:** 2012-02-22

**Authors:** Aziz Ouarssani, Fouad Atoini, Fatima Ait Lhou, Mustapha Idrissi Rguibi

**Affiliations:** 1 Service de Pneumologie, Hôpital Militaire Moulay Ismail, Meknès, Maroc; 2 Service de chirurgie thoracique, Hôpital Militaire Moulay Ismail, Meknès, Maroc

**Keywords:** Tuberculose sternale, abcès, anti-bacillaire, lyse osseuse, Maroc

## Abstract

La tuberculose ostéoarticulaire touche surtout le rachis. L’atteinte sternale est rare, elle représente moins de 1% des ostéomyélites tuberculeuses. L’abcès froid compliqué par une fracture pathologique constitue le mode de révélation le plus fréquent. Le traitement chirurgical assure l’évacuation des abcès et permet le diagnostique histologique et bactériologique. On rapporte deux cas de tuberculose sternale âgés respectivement de 45 ans et de 10ans, colligés au service de pneumologie, l’examen histologique et bactériologique ont permis d’affirmer le diagnostic de tuberculose, et le traitement antibacillaire a permis une évolution favorable .A la lumière de ces observations, les auteurs proposent de faire une mise au point sur l’ostéite sternale tuberculeuse.

## Introduction

La tuberculose ostéoarticulaire représente 1 à 3 % des atteintes tuberculeuses, elle prédomine au niveau du rachis. L’atteinte isolée du sternum est rare, représentant moins de 1% des ostéomyélites tuberculeuses. Elle touche souvent l’adulte jeune vivant dans les régions d’endémie tuberculeuse. son diagnostic est souvent difficile et repose sur l’analyse anatomopathologique des tissus et/ou des prélèvements bactériologiques. Nous rapportons deux cas de tuberculoses sternales chez deux patients âgés respectivement de 45 ans et de 10 ans avec une revue de la littérature.

## Patients et observations

### Observation n°1

**Figure 1:**
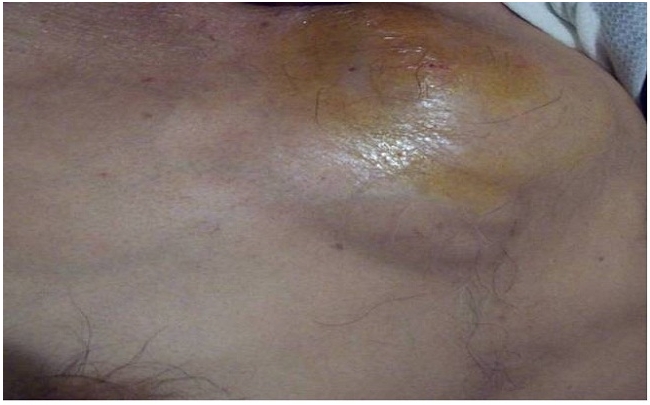
Masse de la paroi thoracique antérieure tendant à la fistulisation

**Figure 2:**
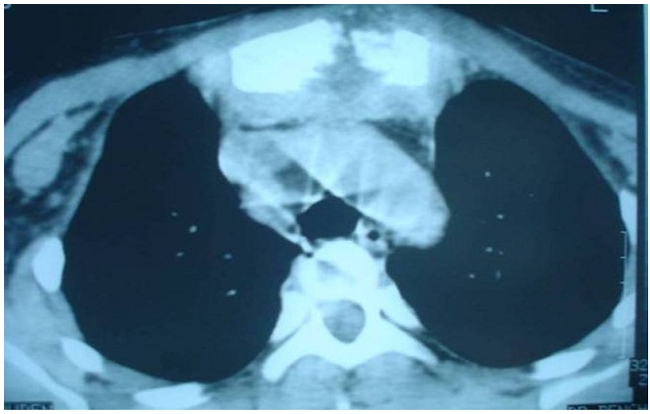
TDM thoracique : remaniement lytique du sternum avec infiltration des parties molles pré et rétro sternal

Mr A G âgée de 45 ans, était hospitalisé dans notre service pour une tuméfaction pré-sternale douloureuse évoluant depuis 2mois dans un contexte d’apyrexie et de fléchissement de l’état général. A l’admission, le patient était apyrétique, pesait 54 kg pour une taille de 1,70 m. L’examen du thorax révélait la présence d’une masse pré-sternale haute, de localisation médiane, mesurant 13 cm de grand axe, douloureuse à la palpation, rénitente, tendant à la fistulisation avec une peau inflammatoire en regard (Figure 1). Le reste de l’examen clinique était sans particularité. La radiographie pulmonaire de face était normale. Une échographie de la masse était réalisée, elle montrait une lésion para sternale gauche, finement hétérogène, male limitée. La TDM thoracique a objectivé un remaniement osseux de type lytique du manubrium sternal avec rupture des corticales antérieure et postérieure associé à une collection en sablier pré et rétro sternal rehaussée en périphérie après injection du produit de contraste (Figure 2). Le bilan biologique notait une vitesse de sédimentation à 55 mm à la première heure, et la numération sanguine montrait une anémie hypochrome monocytaire avec une hémoglobine à 9g/100ml et une hyperleucocytose à 12600 éléments par millimètre cube à prédominance neutrophile. L’intradermo-réaction (IDR) à la tuberculine était positive à 25 mm. La sérologie VIH était négative. La ponction de cette masse à l’aiguille avait ramené un liquide blanc nacré contenant du bacille de Koch à l’examen direct. Le patient avait bénéficié d’une mise à plat avec curetage du foyer osseux, l’étude anatomopathologique des prélèvements avait conclu à une tuberculose caséo-folliculaire évolutive. Un traitement anti-bacillaire était débuté avec deux mois de trithérapie (RHZ) associant la rifampicine® 600mg par jour, l’isoniazide(H) 300 mg par jour et la pyrazinamide (Z) 2000 mg par jour et sept mois de bithérapie (RH). Le patient est revue à la fin du 2 mois du traitement, la cicatrice était propre, et la VS à 8 mm à la première heure.

### Observation n°2

**Figure 3:**
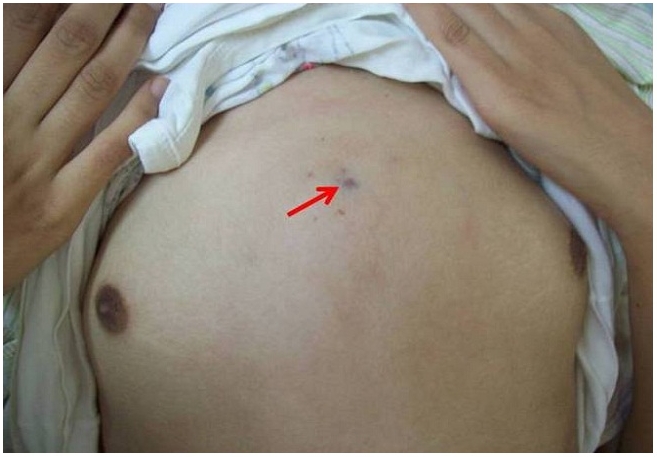
Masse sternale rénitente chez un patient atteint de tuberculose sternale

**Figure 4:**
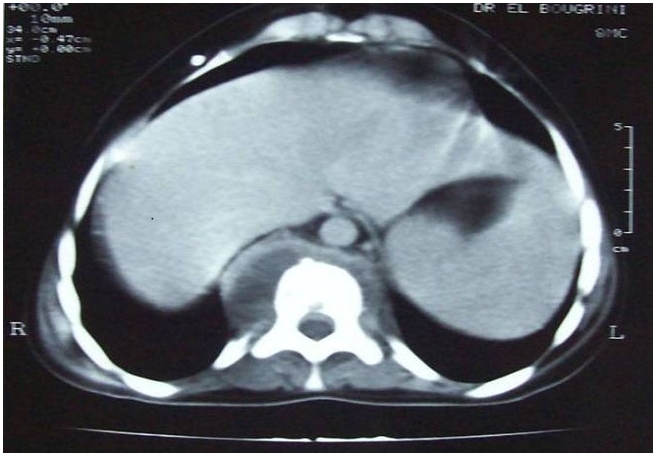
TDM thoracique montrant une lyse sternale avec abcès para vertébral

Une jeune fille âgée de 10 ans, sans antécédents pathologiques particuliers, était admise dans notre formation pour une tuméfaction sternale douloureuse évoluant depuis 3mois dans un contexte d’amaigrissement chiffré à 6Kg. A l’examen, la patiente pesait 27 Kg, la tuméfaction sternale était rénitente, mesurait 6 cm, d’allure non inflammatoire tendant à la fistulisation (Figure 3). La radiographie standard montrait un fuseau para vertébral étendu de la 10éme à la 12éme vertèbre thoracique. Le scanner thoracique retrouvait une lyse osseuse du manubrium sternal et une infiltration des parties molles en regard, associé à des remaniements osseux du corps vertébral de la 10éme vertèbre thoracique et épaississement des parties molles para vertébrales. Le canal rachidien et le mur postérieur étaient respectés. (Figure 4). Le bilan biologique retrouvait un syndrome inflammatoire. L’IDR à la tuberculine était positive. La patiente bénéficiait d’une mise à plat avec curetage du foyer osseux sternal. L’étude histologique des prélèvements confirmait le diagnostic de tuberculose en montrant l’existence de lésions de granulome à cellules géantes avec nécrose caséeuse. Elle était mise sous traitement anti bacillaire associant la rifampicine 300mg par jour, l’isoniazide 150 mg par jour et la pyrazinamide 750 mg par jour pendant 6 mois. L’évolution clinique et biologique était favorable.

## Discussion

La tuberculose osseuse reste toujours une maladie d’actualité, mais son caractère épidémiologique, clinique et évolutif ne cesse de se modifier avec le temps. La tuberculose ostéo-articulaire représente 3 à 5 % des cas de tuberculose et 15 % des tuberculoses extra pulmonaires. Elle prédomine au niveau du rachis et des articulations portantes. La localisation sternale est très rare, Les cas rapportés dans la littérature sont très peu nombreux. Elle représente 0,3 à 1,8 % des cas des tuberculoses ostéo-articulaire [1]. Elle est plus fréquente chez les toxicomanes et les immunodéprimées [2,3]. Cliniquement, elle se manifeste par une tuméfaction des parties molles en rapport avec un abcès froid. Une fracture spontanée sur tuberculose sternale était décrite [4]. L’imagerie est intéressante pour faire le bilan lésionnel et le bilan d’extension. Souvent cet aspect radiologique, comportant une lyse osseuse et une infiltration des paries molles, n’est pas spécifique et fait discuter une tumeur osseuse maligne primitive (sarcome d’Ewing, chondrosarcome), secondaire ou une hémopathie maligne. Le diagnostic est bactériologique et ou histologique. Le traitement est médicochirurgical. Le traitement chirurgical consiste en une mise à plat, évidement de l’abcès et curetage osseux. Le traitement médical repose sur l’association de plusieurs antibacillaires. Les protocoles thérapeutiques courts sont de plus en plus préconisés et aboutissent à la guérison, sans séquelles notamment quand ils sont administrés précocement. Une surveillance clinique, biologique et radiologique est systématique. Elle permet le suivi de l’évolution de la maladie, d’évaluer l’efficacité du traitement et le dépistage d’éventuelles complications [5].

## Conclusion

La tuberculose sternale, bien qu’elle soit rare, doit être évoquée devant des lésions lytiques associées ou non à des collections des parties molles. Le diagnostic de certitude se fait par l’étude histologique et bactériologique. La mise à plats des lésions et le traitement antibacillaire assurent la guérison.
